# Compensation of Verdet Constant Temperature Dependence by Crystal Core Temperature Measurement

**DOI:** 10.3390/s16101627

**Published:** 2016-09-30

**Authors:** Slobodan J. Petricevic, Pedja M. Mihailovic

**Affiliations:** School of Electrical Engineering, University of Belgrade; 11000 Belgrade, Serbia; pedja@etf.rs

**Keywords:** Faraday effect, fiber optic current sensor, temperature compensation, optical activity

## Abstract

Compensation of the temperature dependence of the Verdet constant in a polarimetric extrinsic Faraday sensor is of major importance for applying the magneto-optical effect to AC current measurements and magnetic field sensing. This paper presents a method for compensating the temperature effect on the Faraday rotation in a Bi_12_GeO_20_ crystal by sensing its optical activity effect on the polarization of a light beam. The method measures the temperature of the same volume of crystal that effects the beam polarization in a magnetic field or current sensing process. This eliminates the effect of temperature difference found in other indirect temperature compensation methods, thus allowing more accurate temperature compensation for the temperature dependence of the Verdet constant. The method does not require additional changes to an existing Δ/Σ configuration and is thus applicable for improving the performance of existing sensing devices.

## 1. Introduction

Fiber optic magnetic field sensors (and current sensors by extension—FOCS) have been under investigation as an important new technology suitable for industrial application at voltage levels ranging from low voltages to hundreds of kilovolts [1,2,3,4]. Advances in electronic processing and the abundance of fiber types ease manufacture and offer suitable connectivity options as required by industry.

The development of FOCS has resulted in two mainstream configurations (intrinsic vs. extrinsic) using two Faraday Effect sensing techniques (polarimetric vs. interferometric) [5]. Extrinsic polarimetric types offer easier construction due to their simplicity and ability to match the Faraday medium (usually a crystal) to any particular task and, unlike the intrinsic type, can be made portable. If the sensing crystal is appropriately chosen, extrinsic FOCS does not suffer from linear birefringence that it is inevitable in a coiled sensing fiber [6].

The temperature dependence of a Faraday medium has been identified as an important issue [7,8,9,10,11,12,13,14] requiring compensation in order for the sensor to attain the required level of precision. The choices of the Faraday crystal and the measurement technique are essential for maintaining the extrinsic FOCS advantages as well as for temperature compensation. The magneto-optical quality (the ratio of the Verdet constant and the absorption coefficient) represents the major characteristic of a Faraday crystal since the sensitivity is a function of it. A Faraday crystal should possess no linear birefringence. Circular birefringence reduces sensitivity but can be used for temperature compensation, as will be shown in this paper. The Δ/Σ measurement technique provides normalization to the light source intensity across the whole spectrum and can be realized in both free space and measurement head [15].

This paper will present a new method for sensing the temperature of the crystal bulk (core) without changing the sensor Δ/Σ measurement setup. Only signal processing changes are required and then only in the processing software in order to extract the crystal temperature and compensate for the temperature dependence on the sensor. Compensation involves a simple correction calculation that considerably improves the accuracy of the sensor.

## 2. Materials and Methods

**Figure 1:**
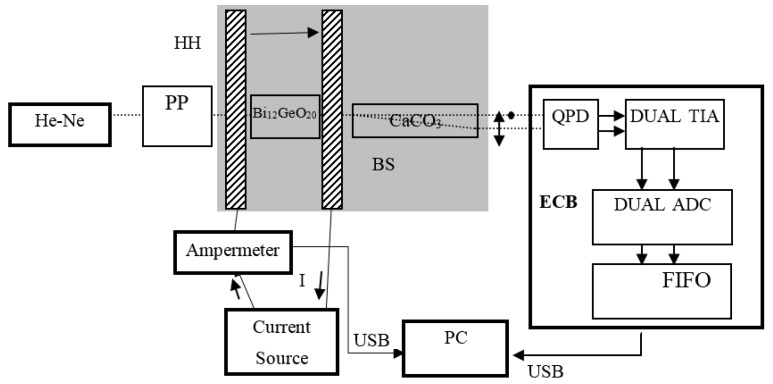
Measurement setup.

Bismuth germanium oxide (BGO) Bi_12_GeO_20_ is a good choice for the Faraday crystal. It possesses no linear birefringence, can be easily grown by the Czochralski technique, and has a large magneto-optical quality. BGO also possesses optical activity. A change of the working temperature of the sensor can reduce sensor sensitivity through temperature dependent optical activity. On the other hand, the optical activity can provide temperature compensation of the sensor output. A BGO crystal in the Δ/Σ measurement configuration and calcite as the beam splitter were used, as depicted in Figure 1. The polarization prism converts polarization fluctuations of the laser to intensity fluctuations that are canceled by the Δ/Σ setup. If the gains of two channels are equalized by matching gains of transimpedance stages and the input polarization is set so that the output polarization in absence of the magnetic field is at 45 degrees with respect to the fast and slow axis of calcite, the output voltages are

(1) $$U_{1}=\frac{kI_{0}}{2}\left(1+sin\left(2\theta \right)\right),\;\text{and}$$

(2) $$U_{1}=\frac{kI_{0}}{2}\left(-sin\left(2\theta \right)\right),$$

where *I*_0_ is the intensity of the light source, and *k* is a constant that includes all optical losses, as well as the optoelectronic conversion efficiency. Thus, the sensor transfer function can be expressed as

(3) $$\theta =\arcsin\frac{\Delta }{\Sigma }=\arcsin\frac{U_{1}-U_{2}}{U_{1}+U_{2}}.$$

It is common practice to express the rotation of the plane of polarization of the light beam (θ) as a function of the sensed quantity, i.e., the sensor transfer function:

(4) θ(T)=V(T)BL,

where *V*(*T)* is the temperature dependent Verdet constant, *L* is the crystal length, and *B* is the magnetic induction component parallel to the light beam. The crystal length is assumed not to be a function of temperature since the coefficient of thermal expansion of a BGO crystal is small with the value of *α* = 16.8 × 10^−6^ K^−1^ [16].

From Equation (4), it is possible to calculate the magnetic field induction,

(5) $$B\left(T\right)=\frac{\theta }{V\left(T\right)L},$$

and determine the sensed current from the known sensor geometry. If *V*(*T)* is not known, *V* is taken to be a constant, and this creates an error in measurement due to temperature variations.

Polarization rotation contains two components: rotation due to the optical activity of the crystal (θ_0_) and rotation due to the Faraday effect θ*_C_*. The proposed setup measures:

(6) $$\theta =\theta_{C}\left(T\right)+\Delta \theta_{0}\left(T\right),$$

where Δθ_0_(*T*) is the output polarization shift from 45°, where the sensitivity is at a maximum. The temperature variations induce changes in the BGO optical activity referenced to the optical activity at the calibration temperature θ_0_(*T*_0_).

(7) $${\Delta \theta }_{0}\left(T\right)=\theta_{0}\left(T\right)-\theta_{0}\left(T_{0}\right).$$

For AC current sensing applications, the temperature and magnetic field are separated in the spectrum of the θ since the current spectrum contains the AC line frequency and its harmonics, whereas the temperature resides in the DC part. The current *I* can be determined as

(8) $$I\left(T\right)=const\cdot \theta_{C}\left(T\right)=const\cdot V\left(T\right)BL.$$

It is possible to measure the temperature of the crystal by measuring Δθ_0_ by Δ/Σ at the moments when the magnetic field induction is zero. There are two such points per period, and further averaging is also possible since the temperature changes slowly. This makes this method inherently capable of good signal-to-noise ratios since averaging the temperature at, say, 100 points (2 points per period, 50 period per second, and 1 reading of temperature per second) significantly improves the SNR.

After the determination of temperature, it is possible to calculate *V*(*T*), and this eliminates the temperature influence on the sensor transfer function.

In other words the temperature can be calculated as

(9) $$T=T\left({\Delta \theta }_{0}\right).$$

Once the temperature of the crystal core is determined, it is possible to compensate the current measurement and obtain the compensated measured current as

$$I_{C}=V\left(B,T\left({\Delta \theta }_{0}\right)\right)=\frac{V\left(T_{0}\right)}{V\left(T\right)}I\left(T\right).$$

In order to calculate the temperature of the crystal core, it is necessary to know the optical activity of the crystal versus temperature. A previous work [17] suggested a linear relation between the polarization plane rotation due to the optical activity θ and temperature in K. The temperature dependence of the Bi_12_GeO_20_ optical activity was measured, and the reported value is 0.0001 rad/mmK = 0.00573 deg/mmK. It is possible to construct a setup that would simultaneously measure the optical activity and the Verdet constant against temperature and thus calibrate the sensor transfer function.

Thus, with knowledge of the temperature dependence of both the optical activity of the crystal and the Verdet constant, it is possible to calculate the Faraday crystal core temperature, calculate *V*(*T)*, and adjust (compensate) the calculated current to make it temperature-insensitive.

The measurement setup can be seen in Figure 1. The light source is a He–Ne laser at 632.8 nm polarized by the polarization prism that is used to set the plane of polarization at the correct position. Faraday crystal (Bi_12_GeO_20_) is next in the optical path causing rotation of the polarization plane due to its own temperature-dependent optical activity and the temperature-dependent Faraday effect. The magnetic field is created by Helmholtz coils (HH) powered from an AC current source with a reference ampermeter connected in series. The relation between the magnetic induction in the coil center and the coil current (*I*) is known; thus, it is possible to measure the Verdet constant. CaCO_3_ is used as a beam splitter producing two coaxial beams with polarization planes set 90° apart. The intensities of the two beams emerging from the BS are sensed using two quadrants from four quadrant photodiodes (QPDs). This is preferred to two individual photodiodes since the quadrants on a quadrant photodiode are more closely matched in responsivity. The photocurrents from the diodes are amplified with transimpedance amplifiers (DUAL TIA) and sampled using a 16-bit dual ADC. The results are relayed to a PC over a USB interface and using a FIFO to prevent data loss. An electronic processing unit (ECB) is encased in a Faraday cage to minimize EMI effects. The temperature of the crystal is controlled by placing the HH and the crystal in an enclosed chamber with temperature control. The chamber is depicted as a gray area in the picture. The chamber contains a temperature measurement unit (thermocouple) for monitoring the crystal temperature.

Measurement begins at a room temperature of 24 °C with the channel gain matching. The PP is set to produce linear polarization at such an angle that the two beams emerging from the BS have equal intensities. This is verified by aligning each beam with both QPD quadrants and measuring both responses. When all four are equal, channel matching is accomplished. A magnetic field is introduced in the coils by the current source, which produces a constant amplitude (about 4 A), 50-Hz sine wave current. The current is monitored by a reference ampermeter and recorded by the PC. The heater is then activated, and the PC records the outputs from both channels while the air in the chamber is slowly heated. This time series is processed by the PC in order to extract peak-to-peak polarization rotation due to the Faraday Effect θ and the crystal’s own optical activity θ_0_. This is realized by calculating the phase of the test current sine wave and using the points in time when the magnetic induction, due to the test current, reaches peaks and zeros. The resulting measurements are recorded to a hard disk for later processing.

## 3. Results

**Figure 2:**
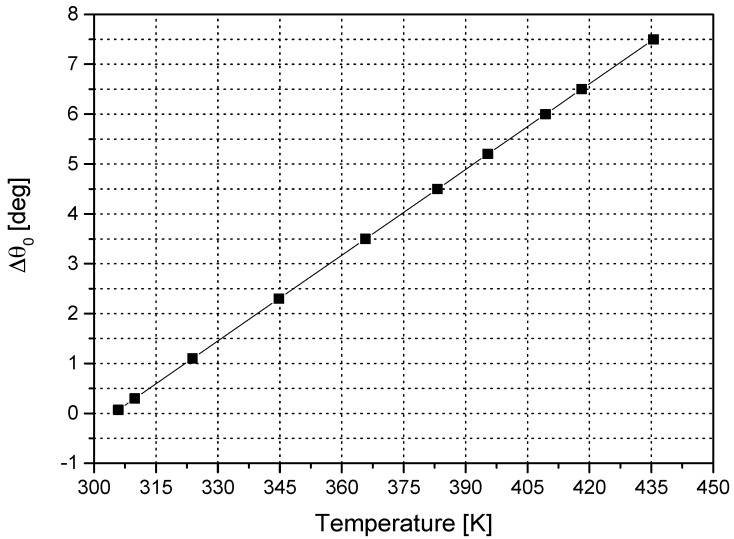
Measured optical activity vs. the temperature of the Bi_12_GeO_20_ crystal.

The dependence of the optical activity of the crystal on temperature is shown in Figure 2.

Using this plot, it is possible to calculate the crystal core temperature and to use the result to compensate *V*(*T*).

**Figure 3:**
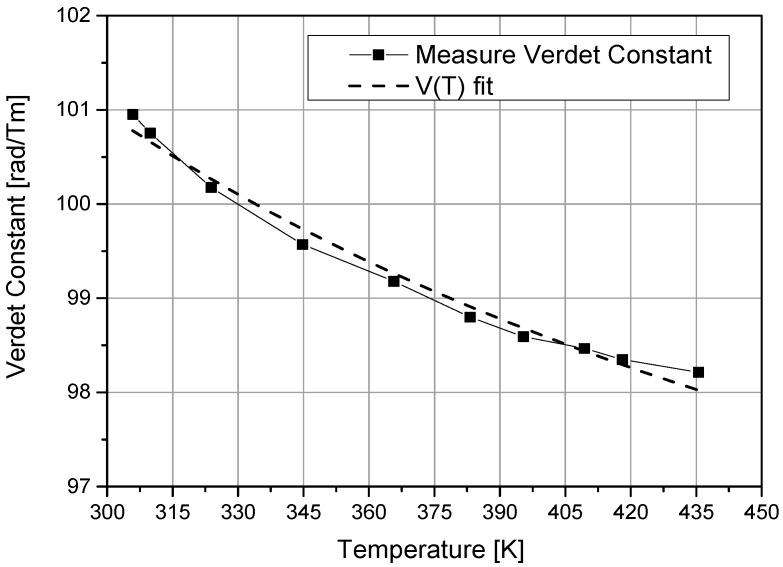
Measured temperature dependence of the Verdet constant of the Bi_12_GeO_20_ crystal.

The variation of the Verdet constant of the crystal with temperature is shown in Figure 3. Verdet constant continually decreases with temperature, falling by 3% within the 24–155 °C range. Earlier research [14] suggested that

(10) $$V\left(T\right)=\frac{A}{T}+B$$

could be used to model the variation. A fit of the experimental data to Equation (10) yields *A =* 2833.89 and *B =* 91.22.

In order to introduce temperature compensation into the measurement, it is necessary to recalculate and thus obtain the compensated sensed current using Equation (11).

(11) $$I_{C}=\frac{101}{\left(\frac{2833.89}{T}+91.22\right)}I\left(T\right).$$

*I_C_* is now the measured Helmholtz coil current obtained from the temperature compensated FOCS.

**Figure 4:**
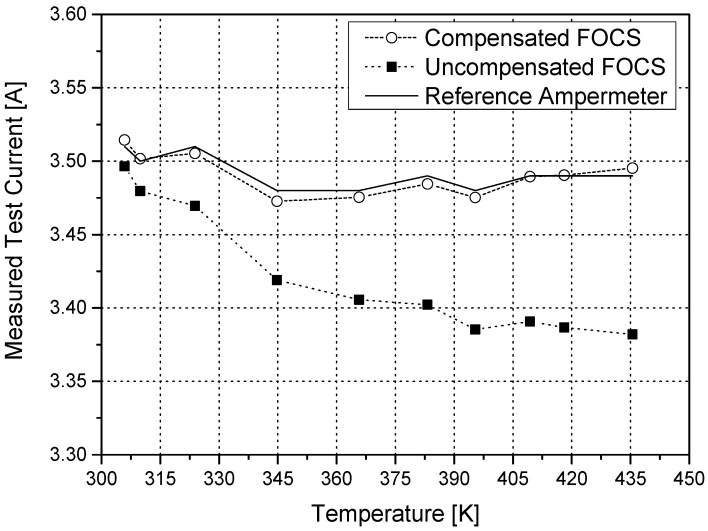
Current in the Helmholtz coils sensed by the reference ampermeter, the uncompensated FOCS, and the temperature compensated FOCS.

Now it is possible to plot the current measurement results obtained from the reference ampermeter, the uncompensated FOCS, and the temperature compensated FOCS (Figure 4).

The uncompensated FOCS underestimates the real current in the coils due to the temperature dependence of the Verdet constant. The temperature compensated FOCS is far more accurate, with the data points grouping around the values registered by the reference ampermeter.

**Figure 5:**
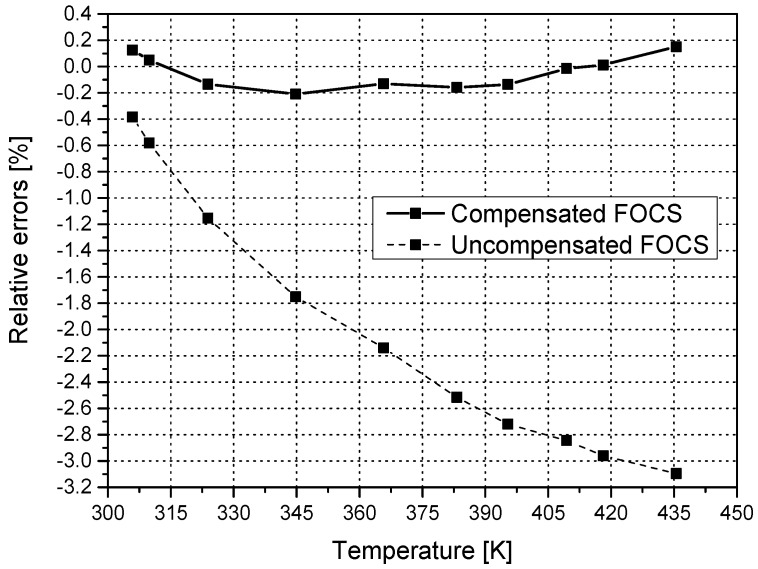
Comparison of relative errors of the uncompensated and compensated FOCS.

The real confirmation of the merit of the compensation method can be seen in Figure 5. This plot clearly shows that the relative error of the uncompensated FOCS matches the variation of the Verdet constant with temperature (around 3%).

## 4. Discussion

The results obtained by temperature compensated FOCS are all located within a margin of ±0.2%, a far better result compared with those obtained with an uncompensated sensor. It is worth noting that the error line crosses zero points twice, due to the application of the theoretically derived equation for the *V*(*T*) fit. It is possible to obtain a much more accurate fit using a polynomial equation, but this kind of fit would require further research into the effects of temperature on the crystal and its modeling.

## 5. Conclusions

Temperature variation in the Bi_12_GeO_20_ crystal used to sense currents is a major factor in the accuracy of the results obtained by FOCS. The methods used to measure the temperature in the immediate vicinity of the sensing crystal may not be accurate enough to compensate the effect of the temperature. By sensing the temperature of the crystal core along the same optical path where the Faraday Effect induces changes in the polarization of the light beam due to the magnetic field, it is possible to compensate the temperature effect. This can be accomplished by measuring the polarization rotation using Δ/Σ at points in time where the magnetic field crosses zeroes and thus requires no changes in the FOCS construction. This solution requires no additional optical elements while offering temperature measurement on the same optical paths where the Faraday rotation is measured. Only some additional computation steps are required, which are easily implemented in a digital signal processing domain, to considerably improve the accuracy of the sensor.

## References

[1] Ulmer E.A. (1990). High-accuracy optical current transducer for electric power systems. IEEE Trans. Power Deliv..

[2] Zubia J., Casado L., Aldabaldetreku G., Montero A., Zubia E., Durana G. (2013). Design and development of a low-cost optical current sensor. Sensors.

[3] Mihailovic P., Petricevic S., Stojkovic Z., Radunovic J. (2004). Development of a Portable Fiber-Optic Current Sensor for Power Systems Monitoring. IEEE Trans. Instrum. Meas..

[4] Petricevic S.J., Stojkovic Z., Radunovic J.B. (2006). Practical application of fiber-optic current sensor in power system harmonic measurement. IEEE Trans. Instrum. Meas..

[5] López-Higuera J.M. (2002). Handbook of Optical Fibre Sensing Technology.

[6] Forman P.R., Jahoda F.C. (1988). Linear birefringence effects on fiber-optic current sensors. Appl. Opt..

[7] Cruden A., Richardson Z.J., NcDonald J.R., Andonovic I. (1995). Optical crystal based devices for current and voltage measurement. IEEE Trans. Power Deliv..

[8] Menke P., Bosselmann T. (1995). Temperature compensation in magnetooptic AC current sensors using an intelligent AC-DC signal evaluation. J. Lightwave Technol..

[9] Perciante C.D., Ferrari J.A. (2005). Faraday current sensor with temperature monitoring. Appl. Opt..

[10] Bohnert K., Gabus P., Kostovic J., Brändle H. (2005). Optical fiber sensors for the electric power industry. Opt. Lasers Eng..

[11] Mihailovic P.M., Petricevic S.J., Radunovic J.B. (2013). Compensation for temperature-dependence of the faraday effect by optical activity temperature shift. IEEE Sens. J..

[12] Williams P.A., Rose A.H., Day G.W., Milner T.E., Deeter M.N. (1991). Temperature dependence of the Verdet constant in several diamaganetic glasses. Appl. Opt..

[13] Yasuhara R., Tokita S., Kawanaka J., Yagi H. Temperature Dependence of the Faraday Rotation of Terbium Gallium Garnet Ceramic. http://www.ile.osaka-u.ac.jp/jp/information/publication/annualreport/2006/6/6.5.pdf.

[14] Barnes N.P., Petway L.B. (1992). Variation of the Verdet constant with temperature of terbium gallium garnet. J. Opt. Soc. Am. B.

[15] Mihailovic P., Petricevic S., Radunovic J. (2006). Improvements in difference-over-sum normalization method for Faraday effect magnetic field waveforms measurement. J. Instrum..

[16] Weber M.J. (2002). Handbook of Optical Materials.

[17] Mihailovic P., Petricevic S., Stankovic S., Radunovic J. (2008). Temperature dependence of the Bi_12_GeO_20_ optical activity. Opt. Mater..

